# Delphi study to develop a multidisciplinary SBIRT strategy for risky drinking in a Flemish community

**DOI:** 10.1186/1940-0640-7-S1-A36

**Published:** 2012-10-09

**Authors:** Leo Pas, Evi Bruyninckx, Hilde de Neyer, Tom Defillet

**Affiliations:** 1 Primary Health Care European Project on Alcohol, Domus Medica, Wezembeek Oppem, Belgium; 2 Flemish Institute for Health Promotion, Flemish Pharmacists Network, Brussels, Belgium; 3 Association for Alcohol and Other Drug Problems (VAD), Brussels, Belgium

## 

A multidisciplinary implementation strategy between general practitioners (GPs), pharmacists, and the mental health sector is needed to effectively implement early interventions for risky drinking in the Flanders region of Belgium. An online Delphi study was set up in three rounds starting with open-ended questions on task views among pharmacists, GPs, and mental health professionals in a single Flemish district. Provisional task definitions, barriers, and suggestions were ordered by profession and send to a larger group of potential respondents throughout Flanders. Respondents were asked to rate importance, acceptability, and feasibility. Responses came from GPs, pharmacists, mental health workers, and pediatricians working in mental health or social services. This allowed us to define subsidiarity of task descriptions and communication principles. General practitioners can offer the full screening, brief intervention, and referral to treatment approach but are in need of structured support and communication with mental health resources more than other professions. Pharmacists are able to detect cases using structured questioning. Brief advice is merely based on indicating the need for further support. Pediatricians and gynecologists see a role for themselves in performing early identification and brief intervention (EIBI) but not in following up; they tend to refer and do not want structured contacts with mental health resources as support. All respondent groups indicate that clear agreements between disciplines need to be in place locally. Case-related discussions and quick diagnostic and therapeutic advice from mental health professionals are also needed. Improved task definitions and communication allow more uptake of EIBI. Principles of EIBI should be included in continuing professional education. Official endorsement and local health promotion is expected to further increase EIBI effects. Web support is planned as an online stepped-care communication platform.

